# Shedding of Microvesicles from Microglia Contributes to the Effects Induced by Metabotropic Glutamate Receptor 5 Activation on Neuronal Death

**DOI:** 10.3389/fphar.2017.00812

**Published:** 2017-11-09

**Authors:** Martina Beneventano, Simona F. Spampinato, Sara Merlo, Mariangela Chisari, Paola Platania, Marco Ragusa, Michele Purrello, Ferdinando Nicoletti, Maria Angela Sortino

**Affiliations:** ^1^Section of Pharmacology, Department of Biomedical and Biotechnological Sciences, University of Catania, Catania, Italy; ^2^Section of Biology and Genetics, Department of Biomedical and Biotechnological Sciences, University of Catania, Catania, Italy; ^3^Department of Physiology and Pharmacology, Sapienza University, Rome, Italy; ^4^I.R.C.C.S. Neuromed, Pozzilli, Italy

**Keywords:** microglia, miRNA, extracellular vesicle, mGlu5, neuroinflammation, CHPG

## Abstract

Metabotropic glutamate (mGlu) receptor 5 is involved in neuroinflammation and has been shown to mediate reduced inflammation and neurotoxicity and to modify microglia polarization. On the other hand, blockade of mGlu5 receptor results in inhibition of microglia activation. To dissect this controversy, we investigated whether microvesicles (MVs) released from microglia BV2 cells could contribute to the communication between microglia and neurons and whether this interaction was modulated by mGlu5 receptor. Activation of purinergic ionotropic P2X7 receptor with the stable ATP analog benzoyl-ATP (100 μM) caused rapid MVs shedding from BV2 cells. Ionic currents through P2X7 receptor increased in BV2 cells pretreated for 24 h with the mGlu5 receptor agonist CHPG (200 μM) as by patch-clamp recording. This increase was blunted when microglia cells were activated by exposure to lipopolysaccharide (LPS; 0.1 μg/ml for 6 h). Accordingly, a greater amount of MVs formed after CHPG treatment, an effect prevented by the mGlu5 receptor antagonist MTEP (100 μM), as measured by expression of flotillin, a membrane protein enriched in MVs. Transferred MVs were internalized by SH-SY5Y neurons where they did not modify neuronal death induced by a low concentration of rotenone (0.1 μM for 24 h), but significantly increased rotenone neurotoxicity when shed from CHPG-treated BV2 cells. miR146a was increased in CHPG-treated MVs, an effect concealed in MVs from LPS-activated BV2 cells that showed *per se* an increase in miRNA146a levels. The present data support a role for microglia-shed MVs in mGlu5-mediated modulation of neuronal death and identify miRNAs as potential critical mediators of this interaction.

## Introduction

Metabotropic glutamate (mGlu) receptors are G protein coupled receptors involved in a variety of functions and processes at the central nervous system (CNS) including synaptic plasticity, neurodevelopment, neuronal excitability, pain, addiction, neurodegeneration, neuroinflammation (Conn and Pin, 1997; Nicoletti et al., 2011). Among the three classes of mGlu receptor subtypes, group 1 mGlu, which includes mGlu1 and mGlu 5 receptors, is highly expressed throughout the brain (Martin et al., 1992; Romano et al., 1995) and mGlu5 receptor shows high density also in the spinal cord (Berthele et al., 1999). They are both Gq-coupled receptors, thus their activation causes increase of intracellular Ca^2+^ and predominate at post-synaptic level where they participate to the modulation of synaptic excitability (Shigemoto et al., 1993; Lujan et al., 1996; Biber et al., 1999). Although initial studies made questionable the function of mGlu5 receptor with a dual role, either neuroprotective or neurotoxic, depending on neuronal type and experimental conditions, it is now clearer that mGlu5 receptor interacts with NMDA receptors and likely exerts a permissive role in NMDA excitotoxicity. Hence, several antagonistic modulators of mGlu5 receptor activity exhibit neuroprotective properties whereas mGlu5 receptor agonists are neurotoxic (Parmentier-Batteur et al., 2014). Among others, mGlu5 receptors are not only expressed in neurons, but also in glial cells (Biber et al., 1999). Interestingly, in the cortex, expression of this receptor subtype is even higher in astrocytes than in neurons (Zhang et al., 2014) and appears upregulated in reactive astrocytes (Aronica et al., 2000; Ferraguti et al., 2001; Dolan et al., 2003; Ribeiro et al., 2017). Cultured microglia also express mGlu5 receptor, and its activation has been reported to be associated with reduced inflammation and neurotoxicity (Byrnes et al., 2009) as well as changes in microglia polarization (Loane et al., 2014). However, again, anti-inflammatory effects secondary to activation of mGlu5 receptor are rather controversial as opposing results with reduction of microglia activation by blockade of group 1 mGlu receptors have also been reported (Hsieh et al., 2012). Surprisingly, mGlu5 receptor has also been suggested to act as an alternative binding site for lipopolysaccharide (LPS), thus mediating activation of microglia toward an inflammatory phenotype (Liu et al., 2014).

Microglia affects neuronal function by the release of several factors, but in more recent years an additional way of intercellular communication has been described in several cell types, including glial cells. Extracellular vesicles, either as microvesicles (MVs) or exosomes that differ in size, content and origin, form under basal conditions, or as in the case of microglia, upon stimulation of the purinergic P2X7 receptor. In particular, microglial-derived MVs, ranging from 100 to 1000 nm in diameter, are shed from the extracellular membrane and play as a cargo for cytokines, RNA, miRNA, etc., to neighboring cells. Although the precise kinetics of this interaction are not completely known, shedding of MVs from microglia is a well-established event (Turola et al., 2012; Prada et al., 2013), reported to mediate neurotoxicity by transferring and propagation of pro-inflammatory cytokines (Bianco et al., 2005), or through modulation of β-amyloid aggregation status (Joshi et al., 2014). Even more, MVs can be detected and are increased in the cerebrospinal fluid of humans and rodents under conditions of brain inflammation, suggesting their role in the development of the disease, by spreading the inflammatory process (Verderio et al., 2012).

On these bases, we decided to investigate whether MVs could take part to the effects elicited by mGlu5 receptor activation in microglia and whether these alternative means of interaction between cells could account for the controversial response observed following activation of this receptor.

## Materials and Methods

### Drugs and Reagents

Cell culture plastics were provided by BD Falcon (Milan, Italy). Media, media supplements, serum, trypsin, and antibiotics, were from Invitrogen Srl (Milan, Italy). (RS)-2-chloro-5-hydroxyphenylglycine (CHPG) Sodium Salt, 3,5-dihydroxyphenylglycine (DHPG), and 3-((2-Methyl-1,3-thiazol-4-yl)ethynyl)pyridine (MTEP), were from Tocris (North Point, United Kingdom). 2′(3′)-*O*-(4-Benzoylbenzoyl)adenosine-5′-triphosphate tri(triethylammonium) salt (Bz-ATP), LPS, rotenone were from Sigma–Aldrich (St. Louis, MO, United States). The following primary antibodies were used: rabbit anti-mGlu 5 (1:500; Millipore, Billerica, MA, United States), rabbit anti-flotillin (1:500; Santa Cruz Biotechnology, CA, United States); goat anti-tumor necrosis factor (TNF) α, (1:100; Santa Cruz Biotechnology), FITC-conjugated mouse anti-TNFα, (1:10; Miltenyi Biotec, Macs, Berisc-Gladbach, Germany).

### Cell Cultures

Immortalized murine microglial BV2 cells were cultured at 37°C, with 5% CO_2_, in Dulbecco’s Modified Eagle’s medium (DMEM) supplemented with 5% fetal bovine serum (FBS), penicillin/streptomycin (100 U/ml-100 μg/ml). For the experiments cells were plated in 35 mm tissue culture dishes (200 k/each). The human neuroblastoma SH-SY5Y cell line was cultured at 37°C with 5% CO2, in DMEM/F-12 supplemented with 10% FBS and penicillin/streptomycin (100 U/ml-100 μg/ml). Cells were plated in 35 mm culture dishes (300 k/each) or in 96 well plates (60 k/well). To achieve BV2 cells conditioned medium (CM), BV2 cells were exposed to either vehicle, CHPG (200 μM), LPS (0,1 μg/ml) or their combination, for 24 h, then washed and incubated with fresh medium for further 6 h. This particular paradigm was chosen in order to allow exposure to treatments for a reasonable time to induce effects before removal so that transferred CM did not contain any drug.

### Shedding of MVs

BV2 cell cultures were treated with CHPG (200 μM, for 24 h), the selective mGlu5 receptor antagonist MTEP (100 μM added 30 min before CHPG), and LPS (0,1 μg/ml, for 6 h). They were then stimulated with Bz-ATP 100 μM, for 20 min at 37° C in Krebs-Ringer-buffer (Sigma–Aldrich) and the supernatant was subjected to differential centrifugations at 4°C: 5 min at 300 × *g* to discard cells and debris (P1 pellet); 20 min at 1200 × *g* to obtain P2 vesicle fraction (P2); 60 min at 10000 × *g* to obtain vesicle population (P3). The resulting pellet corresponding to MVs (P3) was resuspended in 30 μl of protein solubilisation buffer MPER (Invitrogen srl) supplemented with protein inhibitors and loaded for gel separation by western blotting analysis. For this specific experiment, the total amount of protein from 8 × 35 mm dishes was resuspended in a small volume and loaded into the gel in order to assess the whole flotillin, expression of MVs formation. Alternatively, MVs were processed for microRNA extraction or resuspended in DMEM/F12 medium and transferred on SH-SY5Y cells. In the latter case four 35-mm dishes per treatment group were used and, after centrifugation, resuspended in a final volume of 1200 μl that were then divided into eight wells of a 96-well multiwell plate (150 μl/well). To achieve experimental consistent conditions, MVs were collected from BV2 cells plated and grown always at the same density and transferred on SH-SY5Y cells also maintained under constant conditions.

### Western Blot Analysis

Protein extraction was performed using the M-PER Mammalian Protein Extraction Reagent (Thermo Fisher Scientific, Grand Island, NY, United States) and protein concentration was determined with the Bradford reagent (Sigma–Aldrich). Forty to 50 μg of protein extract for each sample were diluted in loading buffer, denatured by boiling at 95°C for 5 min. In the case of proteins extracted from MVs, an equal volume of the extracts was subjected to electrophoresis. Polyacrylamide-SDS gel at 10% or 4–15% were used. The proteins were then transferred onto nitrocellulose membranes (Hybond ECL, Amersham) through a semi-dry transfer apparatus for 60 min at 0.8 mA/cm^2^. After transfer, membranes were blocked with Odyssey blocking buffer (LI-COR Biotechnology GmbH, Bad Homburg, Germany) diluted 1:1 with phosphate-buffer, and then incubated with primary antibodies overnight, at 4°C. Immunodetection was performed using specific fluorescent IRDye©680 or IRDye©800-conjugated secondary antibodies (LI-COR). Detection of specific bands was carried out using the Licor Odyssey© infrared Imaging System (LI-COR). Band intensity was analyzed using the image processing software “ImageJ” developed by NIH and in public domain.

### Cell Viability Assay

SH-SY5Y cell cultures were exposed to rotenone in the presence of either BV2-derived CM or MVs derived from BV2-treated cells. To test SH-SY5Y cell culture viability, 3- (4,5-dimethylthiazol-2-yl) -2,5-diphenyl tetrazolium bromide (MTT) assay was performed after 18 h treatments. Cells were exposed to MTT solution (5 mg/ml) for 90 min at 37°C. DMSO was added to dissolve formazan crystals. The absorbance was measured at 540 nm using Varioskan TM LUX multimode microplate reader (ThermoFisher, Milan, Italy).

### Flow Cytometry

After treatments BV2 cells were harvested, fixed in PFA 4% and then washed and centrifuged at 300 × *g* for 10 min. Cells were incubated for 20 min at RT with 300 μl of Inside Stain Kit (Miltenyi Biotec) and then stained with FITC-conjugated anti-TNFα (1:10, Miltenyi Biotec) according to the manufacturer’s instruction for 15 min, at RT. Cells were then washed and resuspended in 20 μl of PBS). Analyses were carried out using Amnis^®^ imaging flow cytometer (Millipore).

### RNA Extraction

RNA extraction from BV2-derived MVs was performed using the RNeasy Micro Kit (Qiagen, Milan, Italy) according to the manufacturer’s instruction. About 20 ng of RNAs were used for reverse transcription (RT) to obtain miRNA-specific cDNAs by using TaqMan^TM^ MicroRNA Reverse Transcription Kit (Thermo Scientific), according to the manufacturer’s instructions. Four-fifths of the cDNA total volumes were analyzed with quantitative real-time polymerase chain reaction (RT–PCR) using TaqMan MicroRNA Assays (Thermo Scientific). RT-PCRs were performed on a 7900 HT Fast Real Time PCR System (Applied Biosystem – Thermo Scientific), by using TaqMan^TM^ Universal Master Mix II, no UNG (Thermo Scientific), according to the manufacturer’s instructions. SnRNA U6 was used as reference gene. Expression fold changes were calculated by the 2^-ΔΔ*C*_T_^ method.

### Reactive Oxygen Species Determination

Determination of reactive oxygen species (ROS) was performed using the 2′,7′-dichlorodihydrofluorescein diacetate (H2-DCFDA) assay (Sigma–Aldrich). SH-SY5Y cell cultures, plated in 96-well plates and treated for 24 h as described in the result section, were loaded with carboxy-H_2_DCFDA at a final concentration of 1 μM and incubated for 30 min in the dark at 37°C, with 5% CO2. After dye’s removal, intracellular fluorescence (excitation 485 nm, emission 530 nm) was measured using Varioskan TM LUX multimode microplate reader. Results are given as fluorescence intensity normalized for the number of viable cells as measured by MTT assay.

### Whole-Cell Patch-Clamp Recordings and Analysis

Cells plated in 35 mm dishes were transferred from cell culture medium to an extracellular recording solution containing (in mM) the following components: 138 NaCl, 4 KCl, 2 CaCl_2_, 1 MgCl_2_, 10 glucose, 10 HEPES, at pH 7.25 with NaOH. Borosilicate patch pipette were pulled with P-1000 puller (Sutter Instruments, United States) and resistance was 3–5 MΩ when filled with an intracellular solution containing (in mM): 115 K^+^ gluconate, 20 KCl, 2 EGTA, 10 HEPES, 2 Mg-ATP, and 0.3 GTP-Na_2_, pH 7.25 adjusted with KOH. P2X7 receptor current was recorded in a low divalent cation extracellular solution to avoid blocking action of Ca^2+^ and Mg^2+^. This solution contained (in mM): 138 NaCl, 4 KCl, 0.1 CaCl_2_, 10 glucose, 10 HEPES at pH 7.25 with NaOH. Bz-ATP (100 μM) was added to this solution and application and removal of P2X7 receptor selective agonist and other drugs (as indicated in figure legends) were made locally by a gravity-driven multibarrel local perfusion system operated by solenoid valves (ValveBank II, Automated Scientific, United States) and controlled by computer. Exchange times were measured by the change in junction currents at the tip of an open patch pipette and were estimated in 126.2 ± 9 ms. Experiments were performed at RT using an EPC7 Plus amplifier (HEKA Elektronik, Germany) in voltage-clamp configuration (holding potential, –70 mV). Data were acquired at 5 kHz and filtered at 1 kHz using a 7-pole Bessel filter and digitized with a low noise data acquisition system, Digidata 1440A (Molecular Devices, United States). Data were recorded and analyzed in pClamp 10 (Molecular Devices) and peak current was normalized to membrane capacitance (Cm) using Microsoft Excel.

### Statistical Analysis

All data are presented as mean ± SEM and are always derived from at least three independent experiments. Data were analyzed by Student’s *t*-test or, where appropriate, one-way ANOVA followed by Neuman–Keuls test for significance. Plots, bar diagrams and statistical analysis were finalized with GraphPad Prism 5 (GraphPad Software, United States).

## Results

**FIGURE 1:**
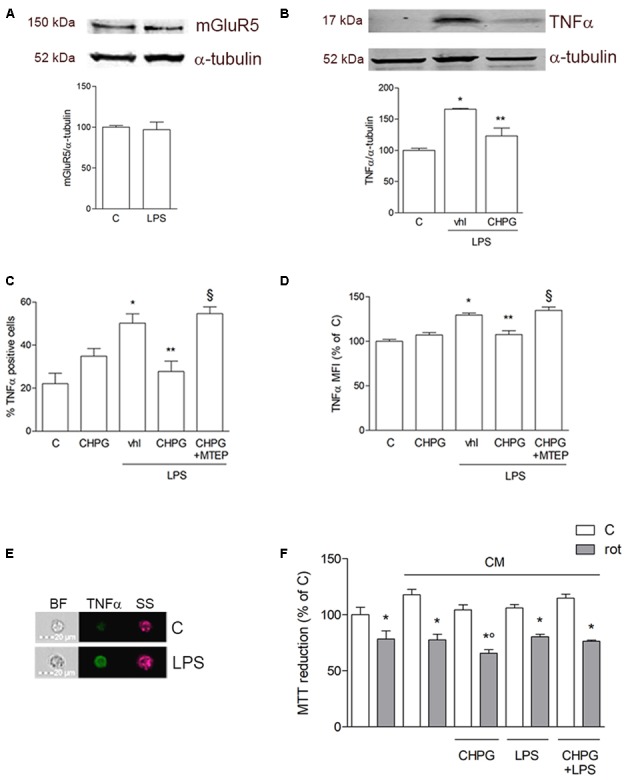
Metabotropic glutamate (mGlu5) receptor mediates anti-inflammatory effect on BV2 microglial cells. Western blot analysis of expression of mGlu5 receptor in BV2 cells treated with LPS (0.1 μg/ml) for 24 h **(A)**. BV2 cells were activated with LPS (0.1 μg/ml for 6 h) after pretreatment with CHPG (200 μM for 24 h) and the selective mGlu5 receptor antagonist MTEP (100 μM added 30 min before CHPG). TNFα expression was evaluated by western blot **(B)** and flow cytometry **(C,D)**. More specifically, assessment of the percentage of TNFα-positive cells **(C)** and the level of fluorescence expressed as mean fluorescent intensity (MFI; **D**) are shown. A representative image acquired by imaging flow cytometry showing cells in their shape and size (brightfield; BF), complexity (side scatter; SS), and TNFα immunostaining (green) is also reported **(E)**. MTT assay on SH-SY5Y cells exposed to rotenone (100 nM for 24 h) in the presence of conditioned medium (CM) from BV2 cells activated with lipopolysaccharide (LPS; 0.1 μg/ml for 6 h) after pretreatment with CHPG (200 μM for 24 h) **(F)**. Student’s *t*-test **(A)** and one-way ANOVA followed by Newman–Keuls test for significance **(B–E)** were applied. ^∗^*p* < 0.05 vs. C; ^∗∗^*p* < 0.05 vs. LPS (Vhl); ^x^*p* < 0.05 vs. CHPG+LPS; °*p* < 0.05 vs. CM rot.

Expression of mGlu5 receptor in microglial BV2 cells was examined by Western blot analysis. As shown in **Figure 1A**, microglial BV2 cells expressed mGlu5 receptor and the protein levels were not modified by activation of BV2 cells after exposure to LPS (0.1 μg/ml) for 24 h. The antinflammatory effect mediated by mGlu5 receptor was confirmed by assessment of TNF-α expression by Western blot and flow cytometry. Treatment for 24 h with the selective mGlu5 receptor agonist CHPG (200 μM) and exposure to LPS (0.1 μg/ml) during the last 6 h, significantly counteracted the LPS-induced increase of TNF-α expression as by Western blot (**Figure 1B**) and by flow cytometry (**Figures 1C–E**) analyses. This specific time point (6 h LPS treatment) was chosen based on published data showing that a significant increase of proinflammatory cytokines (Minogue et al., 2012) and of FoxP3 (Chung et al., 2010) is observed within 6 h following LPS exposure in primary microglia and in BV2 cells, respectively. Specificity of this response was shown by prevention of the effect by the mGlu5 receptor antagonist, MTEP (100 μM), added 30 min before the agonist (**Figures 1C,D**). Changes induced by mGlu5 receptor stimulation in microglial cells modified the response of SH-SY5Y cells to a non-specific toxic insult such as the mitochondrial electron transport inhibitor, rotenone. Transferring of CM from CHPG-pretreated BV2 cells significantly increased SH-SY5Y neuronal death induced by rotenone (100 nM for 24 h; **Figure 1F**). This effect was observed only with CM from quiescent microglia, but not after activation by exposure to LPS (0.1 μg/ml).

In order to dissect the large discrepancies existing in literature regarding the real role of mGlu5 receptor in neuroinflammation, we decided to investigate whether modulation of mGlu5 receptor could also affect shedding and content of MVs involved in the crosstalk between microglia and neurons. To this purpose, BV2 cells were exposed to the ATP stable analog Bz-ATP (100 μM), a P2X7 receptor agonist, for 20 min, a condition that causes blebbing of cell membrane and ensuing formation of MVs (Antonucci et al., 2012).

**FIGURE 2:**
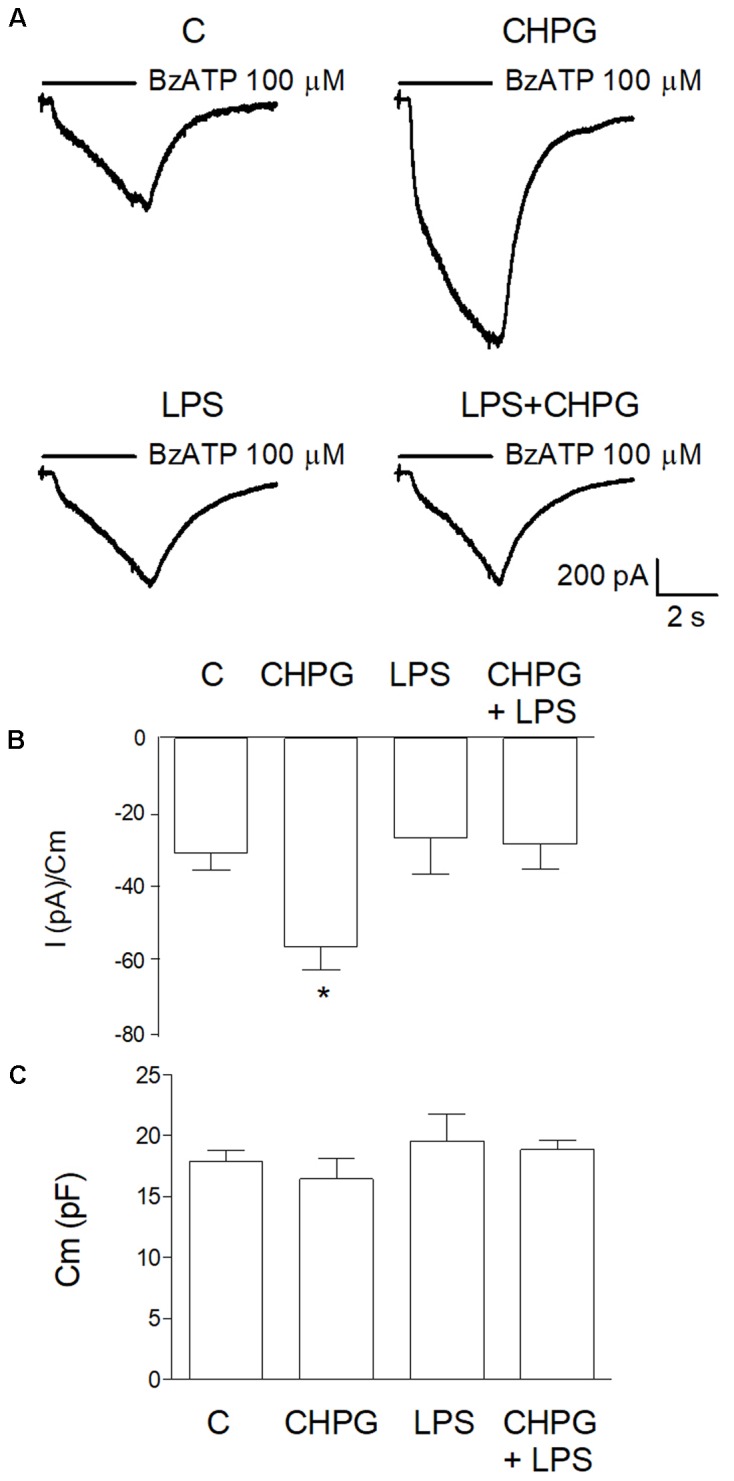
CHPG treatment increases purinergic current through P2X7 receptors in basal but not LPS-activated conditions. In **(A)**, representative traces of purinergic current recorded in BV2 cells in the following conditions: basal (C), after 24 h treatment with CHPG 200 μM, after 6 h treatment with LPS 0.1 μg/ml and after co-treatment of CHPG for 24 h and LPS added during the last 6 h. Currents were elicited after stimulation of P2X7 receptors with the selective agonist Bz-ATP, 100 μM. Summary of purinergic current elicited from cells stimulated as in **(A)** are reported in **(B)**. Data are presented as current (pA) obtained by each cell and normalized to membrane capacitance (Cm), an estimation of cellular surface area. Bars are mean ± SEM (*n* = 11 for C; *n* = 8 for CHPG; *n* = 9 for LPS; and LPS + CHPG), ^∗^*p* < 0.05 by one-way ANOVA followed by Newman–Keuls *post hoc* test. Summary of Cm measured for each cell in different conditions **(C)**. Bars are mean ± SEM of corresponding cells used for bar diagram in **(B)**.

Before assessment of MVs formation, patch-clamp recording was performed in order to test purinergic current through ionotropic P2X7 receptors. BV2 cells exposed to vehicle or CHPG (200 μM) for 24 h were stimulated with Bz-ATP (100 μM) for 3 s in a solution with low divalent ions (0.1 mM Ca^2+^, 0 mM Mg^2+^) to avoid blocking action on channels (Norenberg et al., 2010). Currents elicited by P2X7 receptor activation were significantly increased in BV2 cells pre-treated with CHPG (**Figure 2A**, upper panel and **Figure 2B**). In contrast, when BV2 cells were activated by exposure to LPS for 6 h, pre-treatment with CHPG (200 μM, for 24 h) did not elicit any significant increase in current (**Figure 2A**, lower panel and **Figure 2B**). LPS *per se* was not able to modify P2X7 currents as well (**Figure 2A**, lower panel and Figure **2B**). Membrane capacitance (Cm) showing no change in cell size following treatments is reported (**Figure 2C**).

**FIGURE 3:**
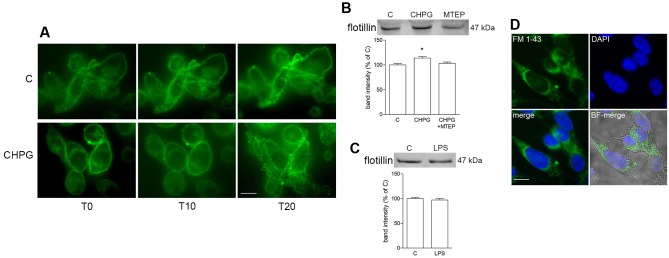
Metabotropic glutamate 5-receptor activation increases microvesicles (MVs) release from BV2 microglial cells. BV2 cells were treated with DHPG (100 μM for 24 h) and labeled with the fluorescent dye FM1-43 (10 μM for 5 min). MVs formation following addition of Bz-ATP (100 μM) was monitored with time-lapse microscopy up to 20 min. Representative images are shown in **(A)**. Western blot of flotillin of equal volume of MVs’ protein extracts from BV2 cells treated with CHPG (200 μM for 24 h) and MTEP (100 μM for 24 h; **B**), or with LPS (0.1 μg/ml for 6 h; **C**). Representative images of MVs shed from FM1-43-stained BV2 cells, transferred on top of SH-SY5Y cultures **(D)**. Nuclei were stained with DAPI. Magnification = 40×**(A)**, 63×**(D)**. Scale bars = 20 μm **(A)** and 10 μm **(D)**. ^∗^*p* < 0.05 vs. control. One-way ANOVA followed by Newman–Keuls test for significance **(B)** and Student’s *t*-test **(C)** were applied to detect statistically significant differences.

Microvesicles shedding from BV2 cells was monitored by time-lapse microscopy following staining of cell membranes with the fluorescent dye FM1-43 (10 μM). MVs formation started soon after addition of Bz-ATP (1 min) and was monitored by sequential captures for up to 20 min (**Figure 3A**). Shed MVs were visible, close to the cell surface for the time points examined. Sequential images did not reveal any eye-detectable change in MVs shedding following mGlu5 receptor activation with the selective agonist CHPG (100 μM) for 24 h (**Figure 3A**). However, expression of flotillin, a protein that is enriched in MVs, was significantly increased in MVs shed from CHPG-treated BV2 cells, when an equal volume, and not an equal concentration, of protein was loaded for gel electrophoresis (**Figure 3B**). This effect recapitulates the increased response to Bz-ATP observed under conditions of mGlu5 receptor activation when P2X7 receptor currents were analyzed, as described above. The increase in flotillin expression was counteracted by the mGlu5 receptor antagonist MTEP (100 μM), added 30 min before CHPG (**Figure 3B**). In contrast, flotillin levels were not changed in LPS-activated BV2 cells (**Figure 3C**). When isolated fluorescent MVs from control and CHPG-treated BV2 cells were transferred on top of SH-SY5Y neuronal cultures, they were rapidly internalized and still visible inside recipient neurons for hours (**Figure 3D**). Pretreatment of BV2 cells with CHPG (100 μM, 24 h) did not modify the interaction of MVs with SH-SY5Y cells as shown by epifluorescent microscopic analysis (not shown).

**FIGURE 4:**
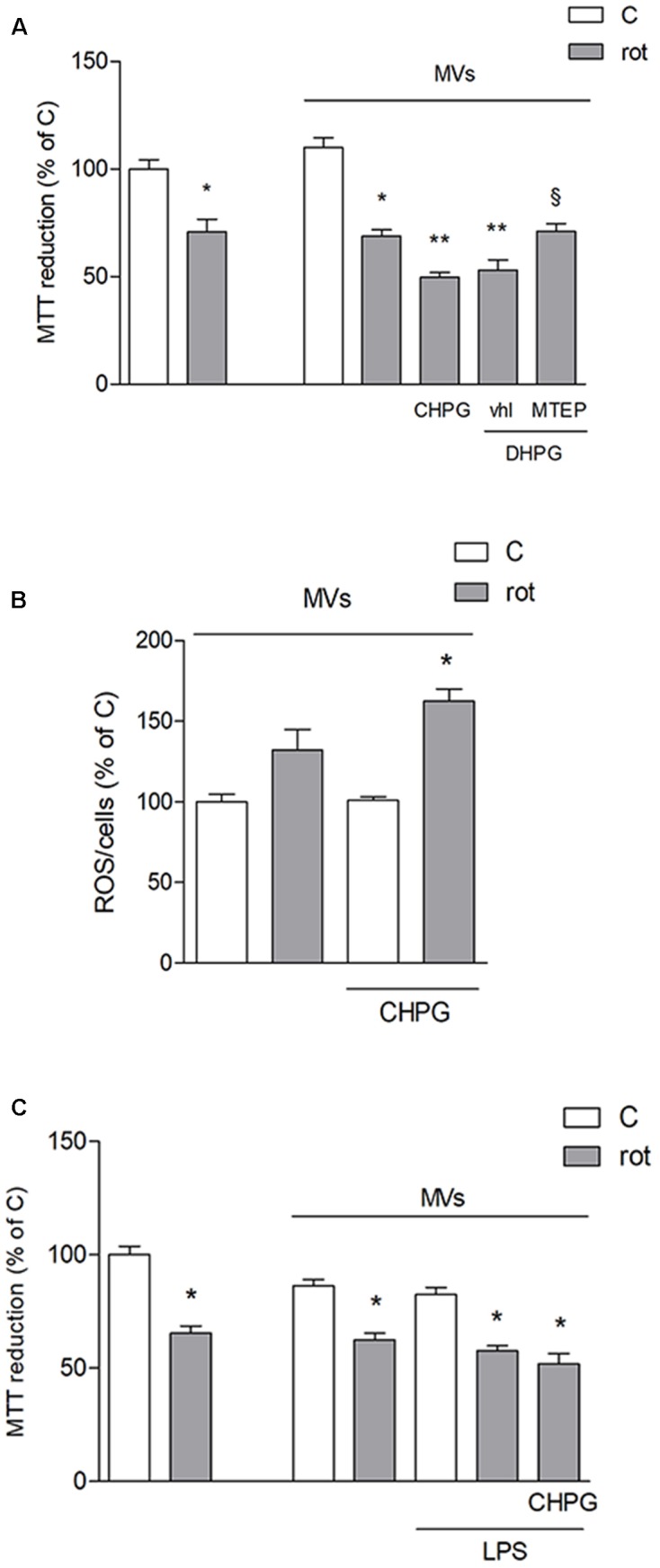
Microvesicles shed from CHPG-treated microglia potentiate rotenone-induced neurotoxicity. In **(A)**, MTT assay on SH-SY5Y cells exposed to rotenone (100 nM for 24 h) in the presence of MVs derived from BV2 cells treated with CHPG (200 μM for 24 h), DHPG (100 μM for 24 h), and MTEP (100 μM for 24 h) and transferred to SH-SY5Y cells after rotenone (100 nM for 24 h) exposure. Determination of ROS levels in SH-SY5Y cells exposed to rotenone (100 nM for 24 h) in the presence of MVs derived from CHPG-treated BV2 cells **(B)**. MTT assay on SH-SY5Y cells (100 nM for 24 h) in the presence of MVs from LPS-activated BV2 cells (0.1 μg/ml for 6 h) pretreated with CHPG (200 μM for 24 h; **C**). ^∗^*p* < 0.05 vs. control; ^∗∗^*p* < 0.05 vs. rot, ^x^*p* < 0.05 vs. DHPG vhl. One-way ANOVA followed by Newman–Keuls test for significance was applied.

**FIGURE 5:**
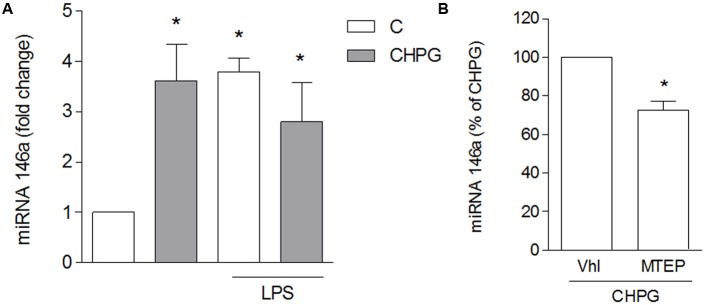
CHPG increases miRNA146a levels in BV2 cells-shed MVs. BV2 cells were activated with LPS (0.1 μg/ml for 6 h) after pretreatment with CHPG (200 μM, total exposure 24 h). MVs were isolated, miRNA extracted and levels of miRNA146a were analyzed by real time PCR **(A)**. In **(B)**, levels of miRNA146a were analyzed in MVs derived from MPTEP pretreated-cells (100 μM added 30 min before CHPG). Results are expressed as percentage of CHPG. One-way ANOVA followed by Newman–Keuls test **(A)** and Student’s *t*-test **(B)** were applied for significance. ^∗^*p* < 0.05 vs. control **(A)**; ^∗^*p* < 0.05 vs. CHPG **(B)**.

To assess the effect of microglia-derived MVs on the viability of neuronal cultures, rotenone was applied to SH-SY5Y cells for 24 h and viability was analyzed by MTT assay. Rotenone, at the low concentration used, (0.1 μM for 24 h) induced about 25–30% neuronal death, either alone or in the presence of MVs from untreated BV2 cells (**Figure 4A**). However, when MVs were from CHPG- or DHPG-treated BV2 cells, a significant increase of rotenone-induced death was observed (**Figure 4A**). This effect was prevented by pre-incubation (30 min) with the selective mGlu5 receptor antagonist MTEP (100 μM) (**Figure 4A**). To analyze whether MVs could interfere with the ability of rotenone to induce oxidative stress, ROS formation was assessed in SH-SY5Y cells exposed to rotenone in the presence of MVs shed from BV2 cells either untreated or exposed to CHPG. In the latter condition, ROS generation appeared significantly increased when compared to rotenone challenge in the presence of MVs from control BV2 cells (**Figure 4B**). Interestingly, potentiation of rotenone toxicity observed in the presence of MVs from CHPG-treated cells was not observed in the presence of MVs shed from LPS-treated microglia (**Figure 4C**). Searching for cellular factors mediating the observed effects and transferred through shed MVs to the neuronal culture, attention was focused on cytokines. Specifically, we tried to detect TNF-α and interleukin-6 in protein extracts from MVs. However, under the experimental conditions used in the present study, no cytokine was measurable, consistently, in MVs protein extracts by Western blot or by flow cytometry (not shown). In contrast, miRNA could be isolated and, following an initial screening, our preliminary results identified miR146a as a potential mediator of the observed effects. Its expression appeared in fact significantly modified in MVs from CHPG-treated BV2 cells. More specifically, exposure to CHPG increased the expression of miR146a in BV2 cell-shed MVs (**Figure 5A**), an effect partially counteracted by pretreatment with MTEP (**Figure 5B**). This effect was significantly modified in LPS-stimulated conditions (0.1 μg/ml for 4 h), where CHPG did not affect the increase of miR146a expression induced by LPS itself (**Figure 5A**).

## Discussion

The present study was undertaken with the primary aim to elucidate the role of mGlu5 receptor in neuroinflammation and its modulatory role in microglia. To this purpose, we used BV2 cells, that, for many aspects including gene transcription, protein production and functional attitude, are superimposable to cultured primary microglia, even under activated conditions that mimic an inflammatory state (Henn et al., 2009; Orihuela et al., 2016). The use of a cell line allows also availability of a great amount of cells, hardly warranted by microglia pure cultures. In addition, looking for MVs, it was crucial to deal with a homogeneous cell population and pure cultures of microglia, although highly enriched (90–95% purity), never reach 100% purity and contaminating astrocytes are also a source of MVs.

The BV2 cell line expresses functional mGlu5 receptors (Loane et al., 2009) and has already been used for the study of the signaling pathway activated by this receptor (Chantong et al., 2014). mGlu5 receptor appears the only group 1 mGlu receptor expressed in microglia *in vivo* and also present in microglia cultures. In contrast, mGlu1 receptor is lacking or barely detectable in cultured microglia (Byrnes et al., 2009), although it has been detected in selected microglia cell populations in multiple sclerosis brain (Klaver et al., 2013). Activation of mGlu5 receptors in primary microglia or in BV2 cell cultures has already been shown to exert anti-inflammatory effect, as revealed by reduction of LPS-induced NO and ROS production, TNFα release, and microglial proliferation (Byrnes et al., 2009; Loane et al., 2009).

In our conditions, we confirmed an anti-inflammatory role for mGlu5 receptor following LPS activation, as suggested by the reduction of the pro-inflammatory cytokine TNFα, by western blot and flow cytometry. However, this anti-inflammatory effect did not produce any neuroprotective outcome, as transferred CM to neuronal cultures was not able to modify neuronal death induced by a low concentration of rotenone. A slight increase of rotenone toxicity was rather observed in neurons exposed to CM from CHPG-treated BV2 cells. This appears in contrast with previous reports showing that CHPG could prevent the neurotoxic effect obtained by co-culturing LPS-activated microglia with neurons (Byrnes et al., 2009). Much higher concentrations of CHPG (20 fold higher) were, however, needed to induce neuroprotection against LPS-stimulated BV2 cell toxicity (Byrnes et al., 2009). Hence, our results indicate the ability of mGlu5 receptor to modify the inflammatory phenotype induced by LPS exposure without, however, any positive outcome on neuronal viability.

Despite the literature and our own observation that indicate anti-inflammatory effect mediated by mGlu5 receptor, when attention is turned to neuropathological conditions such as Alzheimer’s (AD) and Parkinson (PD) diseases, in which activated microglia accumulate around brain lesions and play a role in the neurodegenerative process, the scenario dramatically changes. *In vivo* studies do not support in fact a protective effect mediated by mGlu5 receptor (Liu and Hong, 2003; Town et al., 2005). More specifically, activation of mGlu5 receptor has been linked to increased synaptotoxicity induced by β-amyloid peptide in AD and is reported to exert detrimental effect in animal models of PD (reviewed in Bruno et al., 2017). All this large body of evidence suggests that the anti-inflammatory effect mediated by mGlu5 receptor on microglia may be vanished by effect of drugs acting on this receptor directly on neurons or that it may be not strong enough or contrasted by other factors to elicit any protective effect on neurons. In an attempt to elucidate this aspect, we tried to investigate the mGlu5-mediated modulation of MVs that have recently gathered increasing attention for their function as an alternative/additional way of cell-to-cell and, as in this case, glia-to-neuron communication (Turola et al., 2012; Verderio et al., 2012; Fruhbeis et al., 2013; Prada et al., 2013; Tetta et al., 2013; Carandini et al., 2015; Cocucci and Meldolesi, 2015). MVs are shed from microglia upon activation of P2X7 receptor with the stable receptor agonist Bz-ATP (Turola et al., 2012; Prada et al., 2013). Our results show that the current elicited by P2X7 receptor activation in microglia is significantly increased following stimulation of mGlu5 receptor with CHPG. This effect is in line with the reported potentiation of NMDA-evoked currents in selected neuronal populations (Awad et al., 2000; Yang et al., 2011) and suggests a greater response of microglia cells in CHPG-stimulated conditions. Accordingly, CHPG treatment is able to induce shedding of a greater amount of MVs, indirectly measured by the expression of flotillin in CHPG-derived MVs, when equal volumes of proteins were loaded. Our microscopic observation instead was not accurate enough to let us appreciate such changes in MVs production by eye. Of note, LPS-activated microglia behaved differently, as no potentiation of NMDA-evoked currents by CHPG was observed. From our data, we cannot derive any explanation for this different behavior, but it is known that microglial LPS- and P2X7-mediated responses are related (reviewed in Kettenmann et al., 2011) and this interdependence can probably account for the modified sensitivity to CHPG-induced effect. According to the increased response induced by CHPG, a greater amount of MVs is formed and, when transferred to neuronal cultures, the toxic effect induced by rotenone, either as cell viability or as ROS formation, is increased.

Microglial-derived MVs have been reported to carry the pro-inflammatory cytokine interleukin-1β (Bianco et al., 2005) that may mediate the interplay with other microglia and neurons to spread and propagate the inflammatory reaction (Verderio et al., 2012; Prada et al., 2013), thus enforcing the neuroinflammatory process (Colombo et al., 2012). We could not observe any change in rotenone-induced neuronal damage in the presence of MVs and this may be related to the amount of transferred MVs or to the ratio MVs:neurons achieved in our conditions. However, when MVs were derived from CHPG-treated microglia, toxicity induced by rotenone was significantly increased, suggesting again that MVs-mediated glia-to-neuron signal can be amplified by stimulation of mGlu5 receptor in microglia.

miRNA are among factors that have gained, and are expected to yield, increasing attention for their role in glia to neuron communication (Su et al., 2016). miRNA transferred through extracellular vesicles, either from microglia or astrocytes, have been shown to affect neuronal viability (Hu et al., 2012; Saba et al., 2012; Mao et al., 2015; Tang et al., 2016; Coleman et al., 2017). We focused our attention on miR146a that is highly expressed in glial cells (Li et al., 2011), is up-regulated by pro-inflammatory signals (Lukiw et al., 2008) and negatively controls several target genes involved in the inflammatory response (Lukiw et al., 2008; Li et al., 2011). In addition, transfection with miR146a mimics has been shown to promote apoptosis in SK-N-SH neurons (Li et al., 2017). We here report that stimulation of mGlu5 receptor in BV2 microglia cells up-regulates expression of miR146a that, upon P2X7 receptor stimulation, can be packaged into shed MVs and diffuse to neighboring cells. Our data are rather preliminary and further studies are needed to identify the real significance of miR146a up-regulation in MVs from CHPG-treated microglia. Interestingly, also LPS-stimulated microglia up-regulates miRNA146a in MVs and, once again, under these activated conditions, the effect of CHPG is blunted. Since this up-regulation is accompanied by different effects on neuronal death induced by CHPG- and LPS-shed MVs, we have to take into account that a greater number of MVs is formed upon CHPG stimulation and that, together with miR146a, other miRNAs or other MVs components can be responsible for changes in the fate of neuronal cells in response to rotenone. In addition, the similar response induced by mGlu5 receptor agonist and LPS points out the common pathway that is potentially activated, suggesting that mGlu5 receptor may function as a mediator of inflammatory processes in microglia, as already reported (Liu et al., 2014).

In our opinion, at least four different conclusions can be drawn from our results. First of all, we confirmed the modulatory role of mGlu5 receptor on neuroinflammation that, however, does not have an obvious, ensuing influence on neuronal survival. Even more important, these effects may involve not only conventional interplay between glial cells and neurons, but also novel mediators represented by MVs shed from microglia that function as a cargo for neuromodulators. Third, in our experimental conditions, MVs are formed after stimulation of ATP and shedding and content of MVs seem to vary depending on microglia level of activation. Finally, miRNA transferred through MVs may contribute to the interaction between microglia and neurons thus representing a novel, targetable mediator of neuroinflammation.

## Author Contributions

MB, SFS, SM, PP performed all the experiments, collected, and analyzed all data. MC performed the electrophysiogical studies. MR, MP carried out the miRNA studies. FN contributed to extension and revision of the ms. MAS designed the experiments and wrote the manuscript with the contribution of all other authors.

## Conflict of Interest Statement

The authors declare that the research was conducted in the absence of any commercial or financial relationships that could be construed as a potential conflict of interest.
